# A Functional Variant in *MicroRNA-146a* Promoter Modulates Its Expression and Confers Disease Risk for Systemic Lupus Erythematosus

**DOI:** 10.1371/journal.pgen.1002128

**Published:** 2011-06-30

**Authors:** Xiaobing Luo, Wanling Yang, Dong-Qing Ye, Huijuan Cui, Yan Zhang, Nattiya Hirankarn, Xiaoxia Qian, Yuanjia Tang, Yu Lung Lau, Niek de Vries, Paul Peter Tak, Betty P. Tsao, Nan Shen

**Affiliations:** 1 Joint Molecular Rheumatology Laboratory of the Institute of Health Sciences and Shanghai Renji Hospital, Shanghai Institutes for Biological Sciences, Chinese Academy of Sciences, Shanghai Jiaotong University School of Medicine, Shanghai, China; 2 Key Laboratory of Stem Cell Biology, Shanghai Institutes for Biological Sciences, Chinese Academy of Sciences, Shanghai, China; 3 Department of Paediatrics and Adolescent Medicine, LKS Faculty of Medicine, University of Hong Kong, Hong Kong, China; 4 Department of Epidemiology and Biostatistics, Anhui Medical University School of Public Health, Hefei, Anhui, China; 5 Lupus Research Unit, Department of Microbiology, Faculty of Medicine, Chulalongkorn University, Bangkok, Thailand; 6 Academic Medical Center, University of Amsterdam, Amsterdam, The Netherlands; 7 Division of Rheumatology, Department of Medicine, David Geffen School of Medicine, University of California Los Angeles, Los Angeles, California, United States of America; University of Texas Southwestern Medical Center, United States of America

## Abstract

Systemic lupus erythematosus (SLE) is a complex autoimmune disease with a strong genetic predisposition, characterized by an upregulated type I interferon pathway. MicroRNAs are important regulators of immune homeostasis, and aberrant microRNA expression has been demonstrated in patients with autoimmune diseases. We recently identified *miR-146a* as a negative regulator of the interferon pathway and linked the abnormal activation of this pathway to the underexpression of *miR-146a* in SLE patients. To explore why the expression of *miR-146a* is reduced in SLE patients, we conducted short parallel sequencing of potentially regulatory regions of *miR-146a* and identified a novel genetic variant (rs57095329) in the promoter region exhibiting evidence for association with SLE that was replicated independently in 7,182 Asians (*P*_meta_ = 2.74×10^−8^, odds ratio = 1.29 [1.18–1.40]). The risk-associated G allele was linked to reduced expression of *miR-146a* in the peripheral blood leukocytes of the controls. Combined functional assays showed that the risk-associated G allele reduced the protein-binding affinity and activity of the promoter compared with those of the promoter containing the protective A allele. Transcription factor Ets-1, encoded by the lupus-susceptibility gene *ETS1*, identified in recent genome-wide association studies, binds near this variant. The manipulation of Ets-1 levels strongly affected *miR-146a* promoter activity in vitro; and the knockdown of Ets-1, mimicking its reduced expression in SLE, directly impaired the induction of *miR-146a*. We also observed additive effects of the risk alleles of *miR-146a* and *ETS1*. Our data identified and confirmed an association between a functional promoter variant of *miR-146a* and SLE. This risk allele had decreased binding to transcription factor Ets-1, contributing to reduced levels of *miR-146a* in SLE patients.

## Author Summary

Genome-wide association studies have identified quite a number of susceptibility loci associated with complex diseases such as systemic lupus erythematosus (SLE). However, for most of them, the intrinsic link between genetic variation and disease mechanism is not fully understood. SLE is characterized by a significantly upregulated type I interferon (IFN) pathway, and we have previously reported that underexpression of a microRNA, *miR-146a*, contributes to alterations in the type I IFN pathway in lupus patients. Here we identified a novel genetic variant in the promoter region of *miR-146a* that is directly related to reduced expression of *miR-146a* and is associated with SLE susceptibility. The risk allele of this variant confers weaker binding affinity for Ets-1, which is a transcription factor encoded by a lupus susceptibility gene found in recent GWAS. These findings suggest that reduced expression of Ets-1 and its reduced binding affinity to the *miR-146a* promoter both may contribute to low levels of this microRNA in SLE patients, which may contribute to the upregulated type I IFN pathway in these patients. To our knowledge, this is also the first piece of evidence showing association between a genetic variant in a promoter region of a miRNA gene and a human disease.

## Introduction

Systemic lupus erythematosus (SLE) is a chronic autoimmune disease with a complex etiology and diverse clinical manifestations [1]. The role of genetic factors in the SLE risk has long been established, and demonstrated in familial aggregations, twin studies, and sibling recurrence rates [2]. Recently, high-throughput technologies have facilitated genome-wide association studies (GWASs) across different populations. This approach, accompanied by large-scale replications, has not only confirmed the association of many established susceptibility genes, but has also presented convincing evidence of novel genetic loci involved in SLE [3]–[8]. As members of the Asian Lupus Genetics Consortium, we have also performed a GWAS in Asian populations and have identified variants in *ETS1* and *WDFY4* that are associated with SLE [9]. A combination of GWAS data from different ethnic groups will clearly provide new insights into the genetics of SLE and further our understanding of the pathogenesis of lupus [10], [11].

To use genomic tools to study the mechanisms of SLE, we and others have independently identified a gene expression signature for lupus patients using microarray profiling [12]–[14], which highlights the pathogenic role of the abnormal activation of the type I interferon (IFN) pathway in human lupus [15]–[17]. Intriguingly, recent investigations suggest a genetic contribution to the variability observed among individuals in the production and signaling of IFN [17], and advances in the genetics of SLE highlight the strong association between the risk of developing lupus and gene variants connected to the production and effects of type I IFN [11], [18].

We recently used a microRNA (miRNA) profiling assay to examine the involvement of miRNAs in SLE, because miRNAs are novel gene expression regulators [19] and important players in shaping the immune response [20]–[22]. This profiling identified a reduction in *miR-146a* expression in lupus patients, and we showed that the underexpression of *miR-146a* contributes to lupus pathogenesis by deregulating the activation of the IFN pathway [23]. However, why *miR-146a* levels are reduced in patients with SLE remains unresolved. *miR-146a* is encoded at 5q33.3. Interestingly, recent data from GWASs in both European and Asian populations have indicated that this region is a novel susceptibility locus for SLE [3], [7], [9], suggesting a plausible role for a genetic variant around *miR-146a* in modulating its expression and thus the disease risk.

Several studies have demonstrated unambiguously that genetic variants in miRNA precursors (pre-miRNA) can affect miRNA expression levels by interfering with the miRNA maturation process and are thus associated with disease susceptibility [24]–[26]. We postulate that genetic variants in both the miRNA promoter and the precursor region may alter mature miRNA production. Given the critical regulatory role of *miR-146a* in the type I IFN pathway and the abovementioned genetic association between this pathway and SLE susceptibility, polymorphisms in the *miR-146a* gene could also potentially confer a disease risk. To assess whether genetic variants modulate *miR-146a* expression and thus contribute to the risk of developing SLE, we sequenced the promoter and key regulatory regions of the *miR-146a* precursor to identify potential functional variants that might be associated with SLE susceptibility. Our subsequent replication and functional studies provide evidence that single-nucleotide polymorphism (SNP) rs57095329 in the *miR-146a* promoter, which affects its mature level, can confer SLE susceptibility.

## Results

### Discovery of SLE-associated *miR-146a* promoter SNPs

*miR-146a* is located at 5q33.3. The transcription start site (TSS) of its primary transcript (*pri-miR-146a*) has been identified [27]. To characterize the essential regulatory region for subsequent genetic analysis, we first cloned *miR-146a* upstream fragments with variable 5′ ends into the pGL3-basic reporter plasmid to analyze its promoter activity. We found that the inclusion of a fragment from nucleotide (nt) −1,091 to nt −611, which contains a known NF-κB-binding site characterized in THP-1 cells [27], was consistently robust to promote luciferase activity in HeLa cells (Figure S1). The inclusion of the more distal region (nt −1,998 to nt −1,091) enhanced neither the basal nor phorbol myristate acetate and ionomycin (hereafter referred to as “PMA+Iono”) -induced activity of the promoter (Figure S1*B*). Therefore, to look for new genetic variants and to characterize their potential association with SLE, we designed four pairs of primers with which to sequence the upstream region that spans the 1,105-bp promoter (nt −1,091 to nt +14) and the consecutive first exon of *pri-miR-146a* (Figure S1*A*), in 360 individuals (180 SLE patients and 180 controls), which served as the discovery panel. We also sequenced the 452-bp region centered on *miR-146a* precursor or exon 2 of *pri-miR-146a*, because it potentially affects mature *miR-146a* production [26], in the same discovery panel. A total of 12 variants were identified, with nine already reported in the dbSNP database Build 130 (Table S1). Five variants had a minor allele frequency (MAF) of >1% (rs17057381, rs73318382, rs57095329, rs6864584, and rs2910164; Figure S1*A*). Therefore, we extended our sequencing analysis to examine these five SNPs in up to 816 patients and 1,080 controls, who were all Chinese Han individuals living in Shanghai. In this expanded study panel, only rs73318382 and rs57095329 showed an association with SLE (Table S2). These two SNPs are separated by 304 bp and are in strong linkage disequilibrium (LD; r^2^ = 0.81; Figure S2). When a Bonferroni correction was applied, the association of rs57095329 with SLE remained highly significant (*P* = 4×10^−4^). Given that rs57095329 is identified through our candidate region sequencing approach and not included in the HapMap database, it is not surprising that this SNP has not been included in commercial SNP arrays. Because published GWASs in SLE of both Asian and European populations detected association signals at rs2431697 and rs2431099 (Figure S3), 15 kb and 8 kb upstream from *miR-146a* TSS, respectively, we extended our genotyping of rs2431697 and rs2431099 using 1,896 Shanghai samples. Both SNPs showed significant association with the disease (Table S2), while rs57095329 produced the best association signal among the three SNPs in the same dataset (Table S2). Therefore, we focused on rs57095329 in the subsequent experiments.

### Replicated association of rs57095329 with SLE in independent cohorts

We replicated the association between rs57095329 and SLE using a TaqMan genotyping assay in another two panels from Hong Kong, China, and Bangkok, Thailand. We also added 1,536 patients from the central China area to our mainland China cohort, and the newly added patients showed an allele frequency for rs57095329 very similar to that in the discovery panel (MAFs of 20.53% and 20.77%, respectively). This replication provided consistent evidence for the association, revealed by an allelic association analysis (Table 1). When all the samples were included (3,968 patients and 3,214 controls in total) to conduct a meta-analysis, there was strong evidence that the minor G allele of rs57095329 conferred a risk of SLE (*P*_meta_ = 2.74×10^−8^, odds ratio [OR] = 1.29, 95% confidence interval [CI] = 1.18–1.40; Table 1). There was no significant difference among the ORs for the three independent cohorts (*P* = 0.33), when the Breslow–Day test installed in PLINK was used [28], although the SNP showed significant allele frequency differences in respective controls. Recessive mode of action seemed to be supported in the Chinese mainland cohort and the cohort from Thailand (OR = 2.47 and 2.11 for the two cohorts, respectively), compared with the allelic OR of 1.35–1.36 for the two cohorts. However, this was not supported by the result for the Hong Kong cohort, where the same OR was observed for both the recessive mode and the allelic test (OR = 1.18), reflecting certain variations among the different cohorts.

**Table 1:** Association between the rs57095329 G allele and SLE in independent cohorts and in the combined sample.

Cohorts	case/control	Frequency		χ^2^	*P* value	OR(95% CI)
		case	Control			
Mainland China	2,352/1,080	0.21	0.16	17.55	2.79E-5	1.35(1.17–1.55)
Hong Kong	1,152/1,152	0.23	0.20	4.88	0.027	1.18(1.02–1.36)
Bangkok	464/982	0.29	0.23	10.99	9.16E-4	1.36 (1.13–1.63)
Meta-analysis					2.74E-8	1.29(1.18–1.40)

We also examined whether the genetic variant is specifically associated with disease risk in patients with lupus nephritis. Although only the discovery panel in the Chinese mainland cohort showed a significant association in a patient-only analysis, a similar trend was also observed in the Hong Kong and Bangkok cohorts, with a marginal *P* value of 0.093 and an OR of 1.105 when patients with nephritis were compared with patients without it (Table S3).

### Association between rs57095329 and *miR-146a* expression

**Figure 1:**
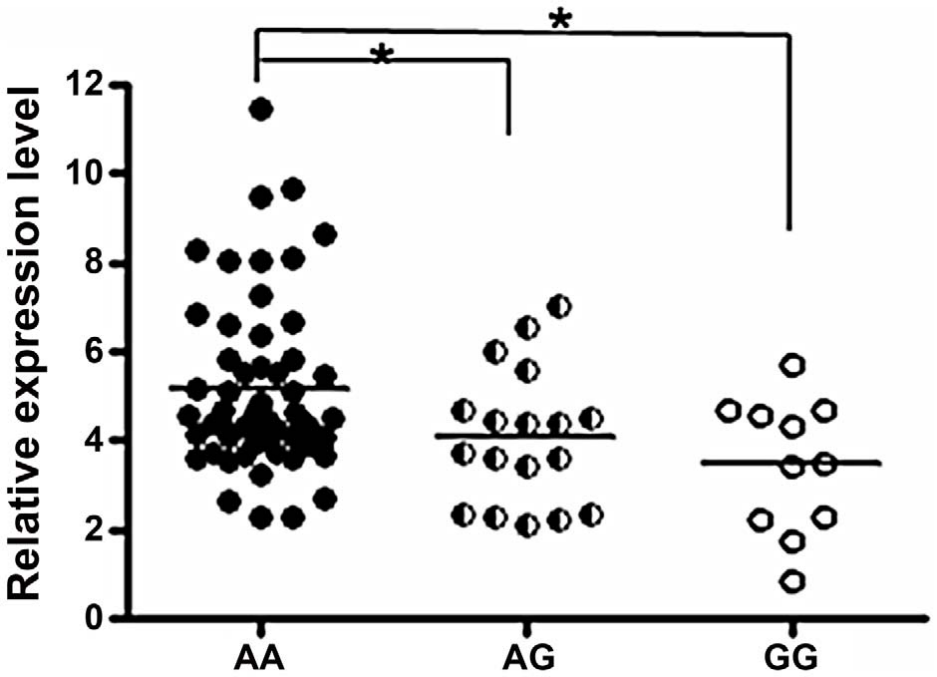
Comparison of *miR-146a* expression levels between groups of healthy individuals with different rs57095329 genotypes. The horizontal line indicates the mean expression level within each group. * indicates *P*<0.05.

We explored the association between rs57095329 and *miR-146a* expression. Mature *miR-146a* levels were determined with a TaqMan microRNA assay in 86 healthy controls with known genotypes and available RNA samples. Compared with individuals with the AA genotype, individuals with heterozygous AG genotype for rs57095329 had lower levels of *miR-146a* (*P* = 0.0438; Figure 1), while individuals with GG genotype had the lowest *miR-146a* levels (*P* = 0.0197; Figure 1). This association indicates that rs57095329, located in the *miR-146a* promoter, may function by regulating the transcription activity and expression levels of *miR-146a*.

### Allelic difference of rs57095329 in *miR-146a* promoter activity

**Figure 2:**
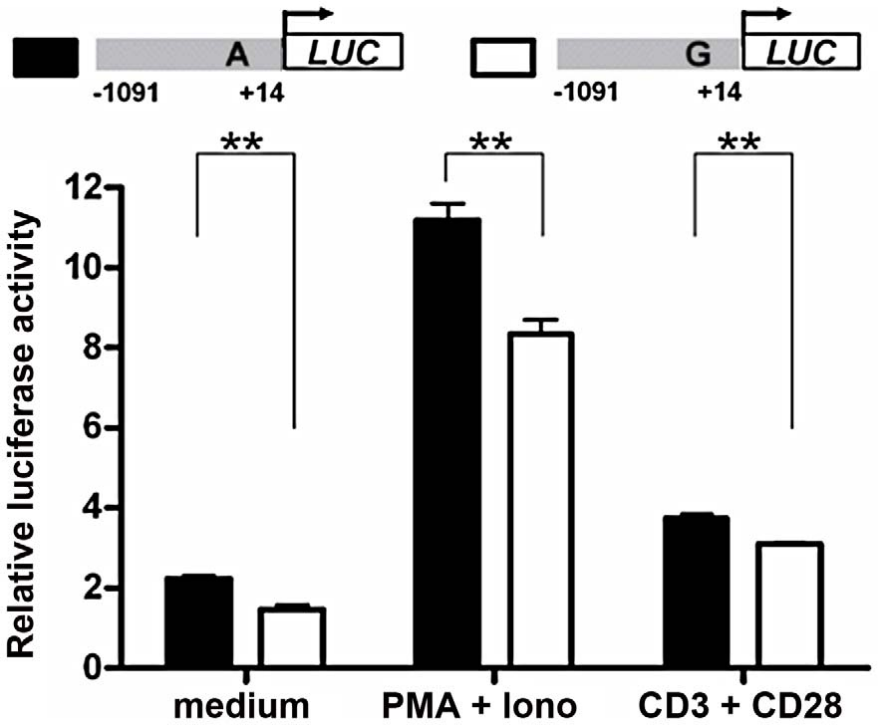
Allelic difference of rs57095329 in *miR-146a* promoter activity. Shown is a schematic representation of reporter gene constructs driven by the full-length *miR-146a* promoter containing one or other of the rs57095329 alleles (*upper*) and the relative luciferase activity of the two constructs in Jurkat cells in both the steady (medium) and activated states (*lower*). For activation, the cells were stimulated with PMA+Iono or with anti-CD3+anti-CD28 antibodies for 6 hours (see Materials and Methods). The data shown are means ± SEM and are representative of three independent experiments performed in triplicate. ** indicates *P*<0.01.

To explore molecular mechanisms of the association between rs57095329 and *miR-146a* expression, we examined whether the variant is functionally significant by altering the *miR-146a* promoter activity. We generated reporter gene constructs containing either rs57095329 allele and transfected different cell lines with the reporter plasmids, so that the effect of each allele on the *miR-146a* promoter activity could be evaluated in the context of the full-length promoter. First, the construct carrying the A allele had higher basal activity in Jurkat T cells than the construct carrying the risk-associated G allele, when a luciferase assay was performed 24 hours after electroporation (Figure 2). This finding is consistent with our previous observation of an association between reduced *miR-146a* expression and SLE disease. Moreover, when the cells were activated by PMA+Iono or anti-CD3 plus anti-CD28 antibodies after transfection, the induced activity of the promoter with the A allele remained higher (Figure 2). Similarly, an approximately 50% reduction in the activity of the promoter with the risk-associated G allele was observed in HeLa cells under both rested and PMA+Iono-activated conditions (Figure S4 *A*). This difference in promoter activity was also consistently found in steady-state Raji B cells and 293T cells (Figure S4 *B* and S4*C*). Considering these data together, our reporter gene assay showed that the disease-associated G allele reduced the promoter activity of *miR-146a*.

### Allelic difference of rs57095329 in nuclear protein–binding activity

**Figure 3:**
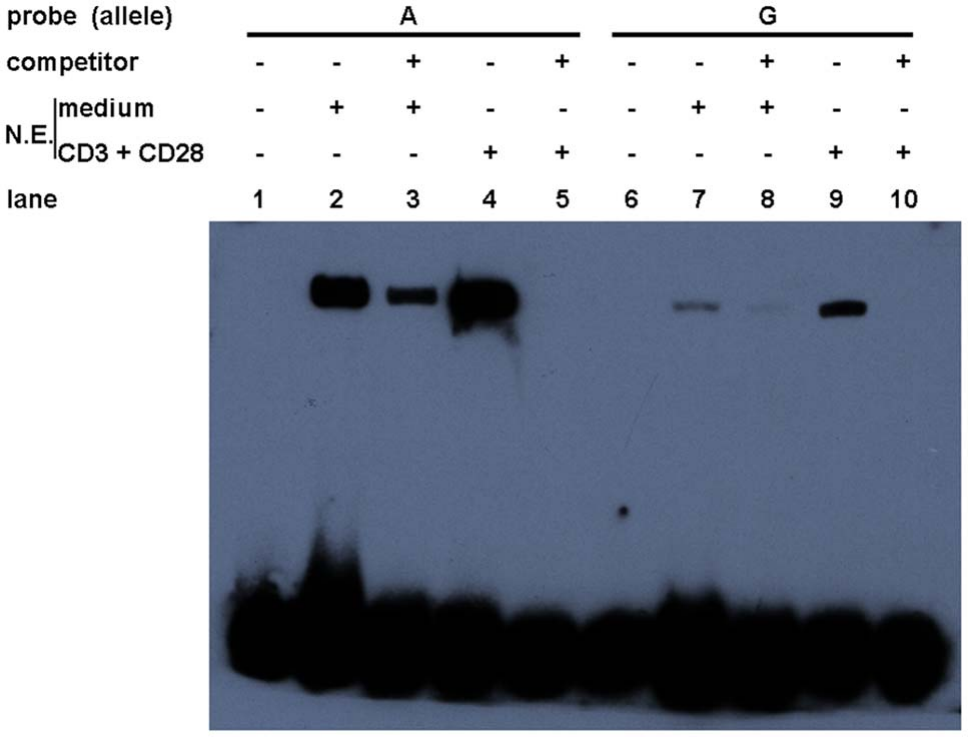
Allelic difference of rs57095329 in binding affinity to nuclear proteins. Shown is a gel-shift assay of the allelic probes (A or G) with nuclear extracts (N.E.) prepared from either rested (medium) or anti-CD3+anti-CD28-activated Jurkat cells. Also shown are the results of a competition assay performed with the addition of 100-fold unlabeled competitor oligonucleotides. The experiment was repeated three times.

To examine whether allelic difference in promoter activity may be attributable to their different binding capacities for nuclear factors, two probes corresponding to the 24-bp *miR-146a* promoter region, centered on rs57095329, were synthesized and biotin-labeled for an electrophoretic mobility shift assay (EMSA), and unlabeled oligonucleotides were used as the “competitors”. Nuclear extracts were then prepared from resting and anti-CD3+anti-CD28-activated Jurkat cells. As shown in Figure 3, probe A formed much more DNA–protein complexes with the nuclear extracts from resting Jurkat cells than did probe G (lane 2 versus lane 7), indicating that the promoter carrying the A allele of rs57095329 binds more robustly to nuclear proteins. Once the cells were activated, both probes were able to bind more nuclear proteins. Similarly, in this case, probe A exhibited much stronger binding than probe G (Figure 3: lane 4 versus lane 9). All the DNA–protein complexes were reduced or abolished by the addition of excessive corresponding “competitor” oligonucleotides, demonstrating the binding specificity. Similar results were observed with PMA+Iono-stimulated Jurkat and HeLa cells (Figure S5).

### Alteration of Ets-1 binding to the *miR-146a* promoter by rs57095329

**Figure 4:**
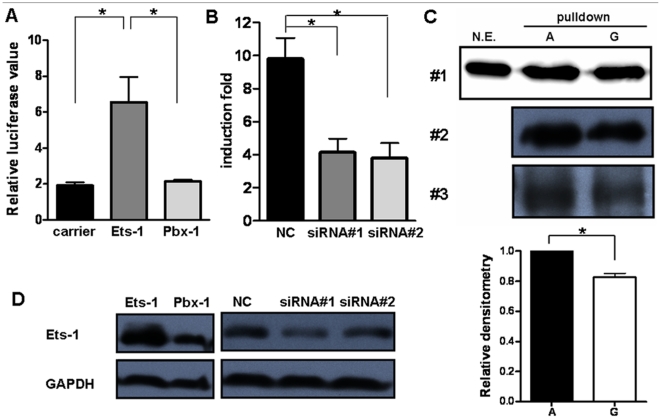
Ets-1 regulates *miR-146a* expression and accounts for the different regulatory activities of the rs57095329 alleles. (*A*) Comparison of the *miR-146a* promoter–reporter gene activities in Jurkat cells after cotransfection with an equal amount of irrelevant carrier vector or *ETS1*- or *PBX1*-expressing vector. The data shown are means ± SEM and are representative of three independent experiments. * indicates *P*<0.05. (*B*) Real-time PCR analysis of the fold induction of *pri-miR-146a* in Jurkat cells after the transfection of *ETS1* siRNA (#1 and #2) or a negative control (NC), followed by cell activation with anti-CD3+anti-CD28 antibodies for 9 hours. The data shown are means ± SEM and are representative of three independent experiments. * indicates *P*<0.05. (*C*) Streptavidin–agarose pulldown assay of transcription factors bound to allelic probes of rs57095329. Biotinylated A or G probes were incubated with nuclear extracts from anti-CD3+anti-CD28-antibody-activated Jurkat cells in the presence of streptavidin–agarose beads. The precipitated proteins were analyzed by western blotting with an anti-Ets-1 antibody (*upper*). The assay was repeated three times. For experiment #1, the nuclear extracts (N.E.) that were used as input for the pulldown assay were also directly blotted as the control. Also shown is a comparison of the relative densitometry values for the blotted bands corresponding to the allelic probes (*lower*). * indicates *P*<0.05. (*D*) Western blot analysis of Ets-1 levels in Jurkat cells after transfection of the same amounts of the indicated expression vectors or siRNA as used in *A* or *B*, respectively. The cells were collected 24 hours or 72 hours after transfection for overexpression or for siRNA-mediated knockdown assays, respectively. GAPDH was used as the loading control.

The findings described above illustrated that rs57095329 alleles conferred differential binding affinity of nuclear extracts to the *miR-146a* promoter. To identify which proteins bind at or near this SNP to regulate the expression levels of *miR-146a*, we performed a bioinformatics search. The Genomatix online tool suggested that the multipotent transcription factor Ets-1 binds to the rs57095329 region (Figure S6). Interestingly, it has been shown that mutation of this predicted Ets-1-binding site resulted in a great reduction in the activity of an *miR-146a* promoter–reporter gene [29]. Here, we performed the following assays and further confirmed the pivotal role of Ets-1 in regulating *miR-146a* expression: the transient expression of Ets-1 greatly enhanced the reporter gene activity from the full-length *miR-146a* promoter in Jurkat cells, compared with that of another transcription factor, PBX1 (Figure 4*A*); knockdown of Ets-1 by small interfering RNA (siRNA) in Jurkat cells directly impaired the induction of *pri-miR-146a* upon T-cell activation (Figure 4*B*); and the overexpression of Ets-1 dramatically enhanced, whereas the knockdown of Ets-1 consistently reduced, the *miR-146a* promoter–reporter gene activity in HeLa cells (Figure S7*A* and S7*B*).

We assessed whether the allelic difference of rs57095329 in regulatory activity is attributable to different binding affinity for Ets-1. We cotransfected different amounts of Ets-1 with the reporter gene construct containing either the A or G allele of rs57095329 into HeLa cells. Increasing the protein levels of Ets-1 greatly enhanced the promoter activity of both constructs. However, the activity ratios of the two constructs (G/A) gradually decreased (Figure S8), suggesting that the inferior ability of the G-allele-containing sequence to bind Ets-1 could be compensated by increasing the levels of Ets-1. To examine the binding affinity more directly, we performed a promoter pulldown assay using streptavidin-conjugated agarose beads. When incubated with nuclear extracts from anti-CD3+anti-CD28-activated Jurkat cells, the biotin-labeled A probe bound more Ets-1 protein than the G probe, as demonstrated by western blotting analysis of total agarose beads precipitated proteins with an anti-Ets-1 antibody (Figure 4*C*). Taken together, these results demonstrate that rs57095329 alters Ets-1 binding, and the risk-associated G allele is less competent than the A allele in the regulation of *miR-146a* expression.

### Interaction analysis of rs57095329 with *ETS1* SNP rs1128334

**Figure 5:**
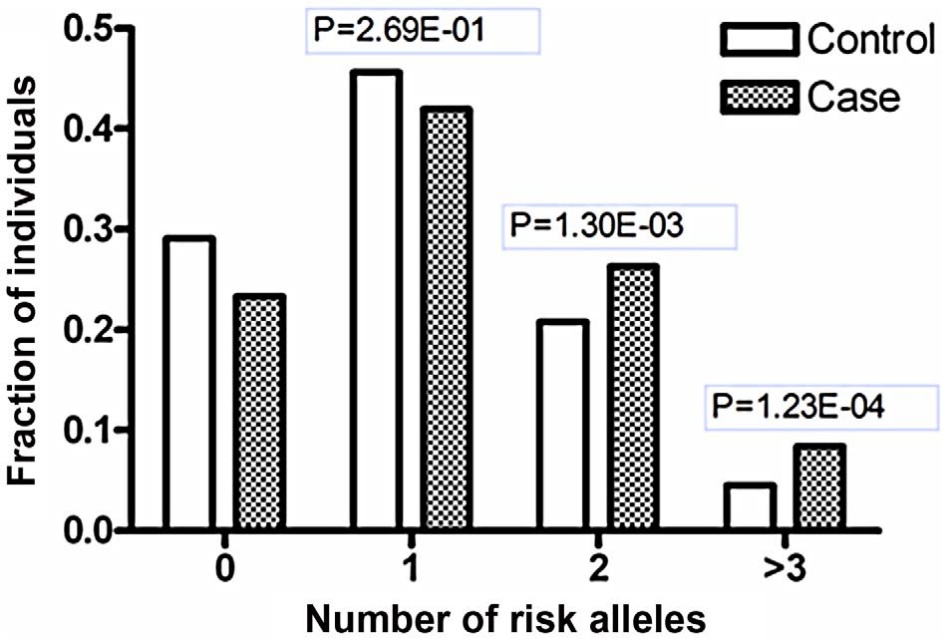
Joint effects of the risk-associated alleles of *ETS1* and *miR-146a*. Shanghai individuals (703 patients and 983 controls) with known genotypes for both variants (rs1128334 in *ETS1* and rs57095329 in *miR-146a*) were analyzed for the distributions of the risk-associated alleles for SLE; fraction of each group with different numbers of risk alleles was shown. The *P* values were calculated with the χ^2^ test for the differences in the allele counts between the patients and the controls in the groups with two or more risk-associated alleles, compared with the groups with zero risk-associated alleles.

It is intriguing that our previous GWAS and that of others identified an association between a functional variant of *ETS1* and SLE [8], [9]. Therefore, we investigated whether there is an interaction between the risk variants of the two genes, rs1128334 in *ETS1* and rs57095329 in *miR-146a*. No epistatic effect was detected between the two variants (*P* = 0.46), when analyzed with a conditional logistic regression test, with the interaction between the two variants treated as a covariate using PLINK [28]. However, we observed additive effects of the risk alleles of *miR-146a* and *ETS1*, suggesting that individuals carrying two or more of these alleles are at greater risk than those carrying only one allele (Figure 5). This additive effect between ETS1 and miR-146a SNPs is also supported by a consistent increase in OR values in analysis of the enlarged samples containing 4,302 individuals with known genotypes for both SNPs (Table S4).

## Discussion

miRNAs have been shown to play an essential role in immune homeostasis, and aberrations in the miRNA-mediated regulation of immune-cell development and function has been linked to autoimmune diseases [30]. In an miRNA profiling study, we recently identified a significant reduction in *miR-146a* expression in lupus patients [23]. Here, we extended this study to determine why the expression of *miR-146a* is reduced in SLE patients. Prompted by the genetic association between the type I IFN pathway and the risk of SLE and by evidence that polymorphic variants in miRNA precursors can modulate miRNA biogenesis and disease risk, we sequenced key regions of *pri-miR-146a* and identified an SLE-associated SNP, rs57095329, within the promoter of *miR-146a*, which functionally affects *miR-146a* expression levels and thus contributes to the risk of SLE. The association of this variant with SLE was consistent in three independent cohorts from mainland China, Hong Kong, and Bangkok (Thailand). Individuals carrying the risk-associated G allele tended to express lower levels of *miR-146a*. To the best of our knowledge, this is the first report of an association between a functional genetic variant in an miRNA promoter and a human disease. It will be interesting to investigate the association between rs57095329 and SLE in other ethnic groups. Another functional variant located in the *miR-146a* precursor, rs2910164, has been associated with cancer development [26], [31], [32], but showed no significant association with SLE in our initial sequencing experiments.

Among the multiple immunological aberrations present in lupus patients, the type I IFN system is thought to play a crucial role in its pathogenesis [15]–[17]. Intriguingly, a number of genes involved in IFN signaling have already been associated with various autoimmune diseases, including SLE [33]. Functional variants in genes encoding key components of the IFN pathway, such as *TYK2*, *IRF5*, and *STAT4*, have been identified and characterized, and their association with SLE has been extensively replicated [34]–[39]. Our recent work characterized the role of *miR-146a* as a negative regulator of the type I IFN pathway by targeting key signaling proteins [23]. Here, the delineation of an SLE-susceptible variant of the *miR-146a* promoter further supports the notion that polymorphic variants linked to IFN pathway molecules contribute to the pathogenesis of lupus. *miR-146a* is embedded in a non-coding RNA with a previously unknown function, so our findings highlight the importance of exploring genetic variants in such regions, which have been more or less ignored in previous genetic studies.

**Figure 6:**
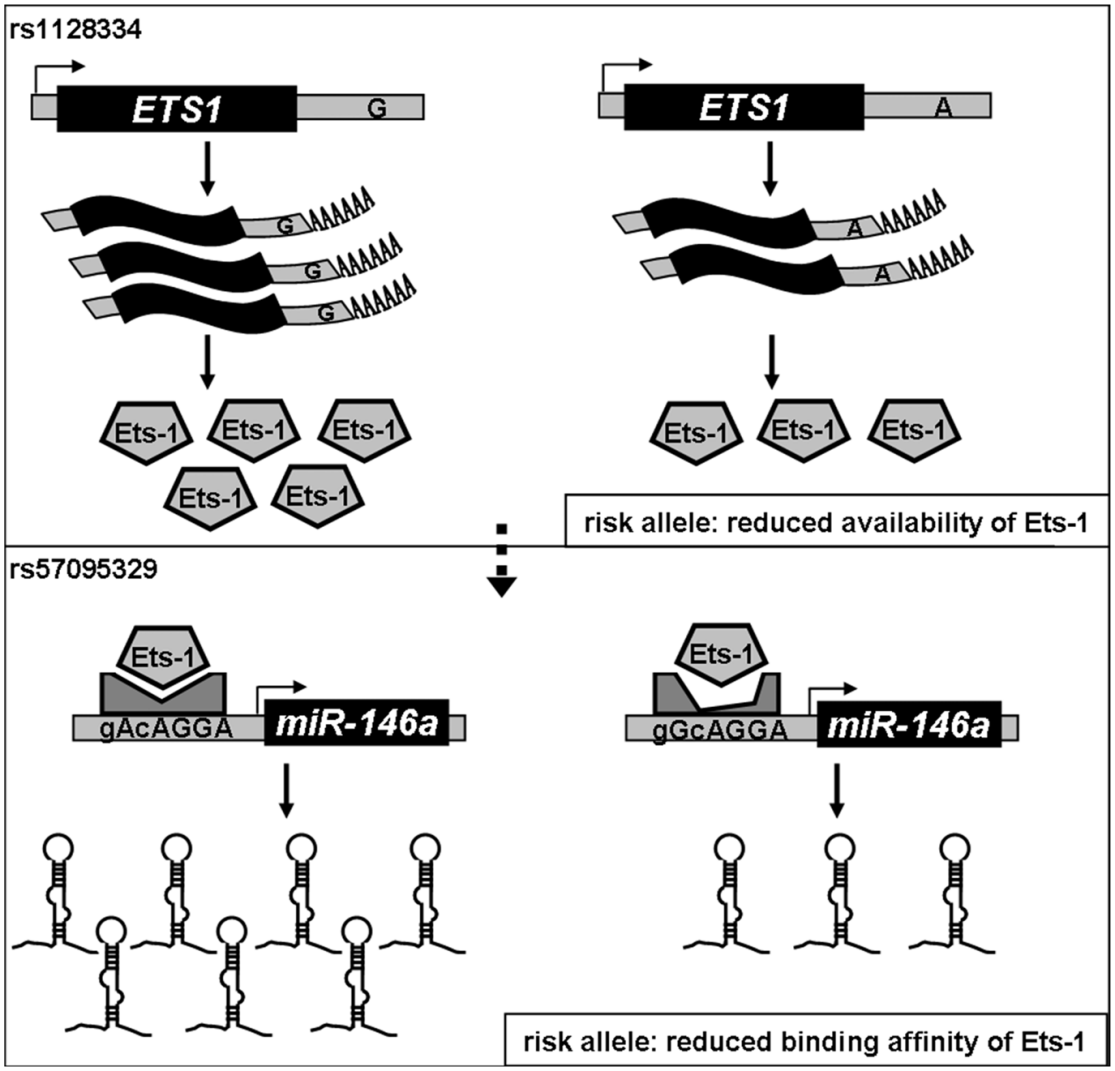
Diagram of the genetic link between *ETS1* and *miR-146a* and genetic contribution to *miR-146a* expression. Expression of cellular *miR-146a* depends on the binding of Ets-1 to the *miR-146a* promoter, so both the availability of and its binding affinity for Ets-1 could affect *miR-146a* expression. We previously found that the A allele of rs1128334 located in the *ETS1* 3′ untranslated region is associated with SLE by reducing Ets-1 expression (*upper panel*). In this study, we show that the binding affinity for Ets-1 is affected by the risk-associated G allele of rs57095329, which causes an incompetent compositional change (*lower panel*), and therefore represents another genetic factor that contributes to the reduced expression of *miR-146a* in SLE patients.

Our findings underline the regulatory role of Ets-1 in *miR-146a* expression, and attribute the allelic difference of rs57095329 to different affinity for Ets-1. Rs57095329 is not located at the core sequence of the Ets-1-binding site (Figure S6), so it only causes an affinity difference, whereas Ets-1 recognition is still well preserved. Nevertheless, the risk-associated G allele of rs57095329 does affect the strongly conserved A residue near the Ets-1 core motif (Figure S6), highlighting the relevance of this SNP. Besides, this is a germ-line regulatory polymorphism and thus potentially functions in each cell type, as reflected in our consistent observation of the reduced activity of a reporter gene carrying the risk-associated G allele in various cell lines (Figure 2 and Figure S4). The attenuation of the promoter activity by the risk-associated G allele of rs57095329 thus accounts, at least partly, for the underexpression of *miR-146a* in lupus patients. Intriguingly, *ETS1* has been characterized as a susceptibility gene for SLE in GWAS results from others and our group [8], [9]. The reduced expression of *ETS1* was shown to be associated with the risk-associated allele of rs1128334 compared with the protective allele, identified in an allelic expression assay [9]. The finding that Ets-1 knockdown led to an inability to induce *miR-146a* expression in vitro (Figure 4*B*) was consistent with reduced miR-146a expression in patients with SLE who have reduced Ets-1 levels. It seems that both rs1128334 in *ETS1* and rs57095329 in *miR-146a* may reduce the expression of *miR-146a*, through the reduced availability of Ets-1 and a reduced binding affinity for Ets-1, respectively. However, we did not detect interaction between the two variants. This may not be surprising because both of these variants only have a quantitative effect on their respective functions. Therefore, an additive effect is observed between the two variants (Figure 5 and Table S4) rather than a strong interaction, which would be the case if both of them totally abolished a function. We propose a working model for the genetic link between *ETS1* and *miR-146a* to illustrate the genetic contribution to the reduced expression of *miR-146a* in SLE patients (Figure 6). We fully appreciate that Ets-1 can modulate a large collection of genes for their expression, and are not trying to limit the contribution of its 3′UTR SNP to SLE solely to affecting miR-146a expression. Yet our functional study, the genotype-expression data, and the additive effect of the two SNPs together provide an interesting connection between these two SLE susceptibility genes.

Recent SLE GWASs identified disease association of two SNPs (rs2431697 and rs2431099) that are upstream of *miR-146a* gene region [3], [7], [9] and our genotyping confirmed their association on our Chinese samples (Table S2). We therefore performed the following analysis to clarify the genetic signals of association across this region: 1) Imputation of rs57095329 into our Asian GWAS dataset, using the individuals of Asian ancestry from the 1000 genome project as the reference panel, suggested that this SNP represented an independent signal (conditional *P* value of 0.90); 2) rs2431099 and rs2431697 was in intermediate LD with each other while they were not in LD with rs57095329 (Figure S2); 3) Conditional analysis indicated that the association of rs57095329 with SLE was independent of those detected at rs2431697 and rs2431099, while the association of rs2431099 with SLE could be attributed to rs2431697 (Table S5); 4) Haplotype analysis showed that rs57095329 and rs2431697 were two independent SLE-associated loci, while rs57095329 had a stronger association in Chinese (Table S6); and 5) There was no correlation between miR-146a expression levels and the genotypes of rs2431697 or rs2431099 (Figure S9). Therefore we have newly identified a relevant SNP (rs57095329) by direct sequencing that the genotyping arrays in GWASs missed due to incomplete coverage, and these SNPs may confer a disease risk through different and independent mechanisms.

In conclusion, our findings add an miRNA gene, *miR-146a*, to the list of SLE-susceptible genes. A genetic variant of the *miR-146a* promoter, rs57095329, is functionally significant in modulating the expression of *miR-146a* by altering its binding affinity for Ets-1.

## Materials and Methods

### Ethics statement

This study was conducted according to the principles expressed in the Declaration of Helsinki. Informed consent was obtained from all subjects. The Shanghai study was approved by the Institutional Review Board of Renji Hospital. The studies of the Hong Kong, Anhui, and Thai samples were approved by the Institutional Review Board of the University of Hong Kong and Hospital Authority, Hong Kong West Cluster, New Territory West Cluster, and Hong Kong East Cluster; the Research Ethics Committee of Anhui Medical University; and the Ethics Committee of the Faculty of Medicine, Chulalongkorn University, respectively.

### Subjects

We recruited 816 SLE patients and 1,080 sex- and age-matched controls, all of whom were from the Chinese Han population in Shanghai, China. Other Chinese mainland samples consisted of 1,536 SLE patients living in central China, collected by collaborators in Anhui province. For the independent replications, samples collected by collaborators in Hong Kong (case vs control: 1,152 vs 1,152) and Bangkok, Thailand (464 vs 982, respectively) were included. All SLE patients fulfilled the American College of Rheumatology (ACR) classification criteria for SLE, and 1,254 patients met the ACR criteria for lupus nephritis.

### DNA sequencing and genotyping

Consecutive overlapping amplicons corresponding to the *miR-146a* promoter region were amplified from genomic DNA extracted from peripheral blood leukocytes. The products were purified and directly sequenced on a 3730 automated sequencer (Applied Biosystems). The 452-bp DNA region around the *miR-146a* precursor was also amplified and sequenced. The primer pairs used are shown in Table S7.

In the replication stage, SNP rs57095329 was genotyped with the specified TaqMan SNP genotyping probes (Applied Biosystems). The assay was run on a 7900HT sequence detection system (Applied Biosystems) and the data were analyzed with the affiliated SDS software, version 2.3. The genotypes of rs57095329 were found to be in Hardy–Weinberg equilibrium (*P*>0.01) in the controls of all three cohorts. The average call rate for all samples was 92%.

### Real-time PCR

Total RNA was extracted from peripheral blood leukocytes or cultured cells using TRIzol (Invitrogen), followed by reverse transcription using a reverse transcriptase kit obtained from Takara. To determine the quantity of *pri-miR-146a*, the cDNA was amplified by real-time PCR with SYBR Green RT–PCR kit (Takara), and the expression of RPL13A was used as the internal control. The primers used are shown in Table S7. To determine the quantity of mature *miR-146a*, the specific TaqMan MicroRNA Assay kit (Applied Biosystems) was used, and the expression levels were normalized to snRNA U6. The assays were performed on a 7900HT real-time instrument (Applied Biosystems). Relative expression levels were calculated using the 2^−ΔΔCt^ method.

### Constructs

To create the *miR-146a* promoter–luciferase reporter constructs, three fragments of variable lengths, corresponding to the upstream region of the TSS of *pri-miR-146a*, were amplified and cloned into the pGL3-basic luciferase vector (Promega). To compare the activities of *miR-146a* promoters containing the different rs57095329 alleles, the full-length 1105-bp fragment was amplified from individual homozygous templates. The ETS1 overexpression vector was a kind gift from Dr Gang Pei, and the PBX1 overexpression plasmid was created by replacing the inserted *ETS1* sequence with the *PBX1* coding sequence. The primers used are shown in Table S7. All constructs were verified by sequencing.

### Cell culture, transfection, and stimulation

Jurkat and Raji cells were grown in RPMI 1640 medium supplemented with 10% fetal bovine serum. These two cell lines were electroporated with 2 µg of the indicated luciferase reporter vector and 0.2 µg of a modified pRL-TK vector, using a nucleofector device (Amaxa). Alternatively, the reporter gene vectors were electroporated in combination with 1.5 µg of an *ETS1*- or *PBX1*-expressing vector. For the knockdown of *ETS1*, 3 µg of *ETS1* siRNA or negative control oligonucleotides (all from GenePharma, Shanghai) were transfected. HeLa and 293T cells were grown in Dulbecco's modified Eagle's medium supplemented with 10% fetal bovine serum. These two cell lines were transfected using Lipofectamine 2000 (Invitrogen), with the *ETS1*- or *PBX1*-expressing vector or *ETS1* siRNA alone, or in combination with 50 ng of the indicated luciferase reporter vector and 5 ng of a modified pRL-TK plasmid. Where indicated, an irrelevant “carrier” vector was added to ensure that equal total amounts of plasmid DNA were transfected among the groups. For cell activation, Jurkat and HeLa cells were stimulated with PMA (100 ng/mL; Sigma) and ionomycin (1 µM; Sigma) for the indicated times. Alternatively, Jurkat cells were activated with plate-bound anti-CD3 antibody (coating solution: 5 µg/mL; eBioscience) and soluble anti-CD28 antibody (2 µg/mL; eBioscience).

### Reporter gene assay

Cells were cultured for 24 hours or 48 hours after transfection with the reporter gene vectors together with the ETS1 expression vector or siRNA, respectively. The cells were then maintained resting or activated for 6 hours and lysed. Their luciferase activity was measured on a luminometer (LB960; Berthold) using the Dual-Luciferase Reporter Assay System (Promega). The ratio of firefly luciferase to *Renilla* luciferase was calculated for each well.

### EMSA

Jurkat and HeLa cells (1×10^7^) were activated or left to rest for 2 hours, and then their nuclear proteins were extracted with a Nuclear Extract Kit (Active Motif), according to the manufacturer's protocol. The protein concentrations were determined with the DC Protein Assay Kit (Bio-Rad). Double-stranded allelic probes were synthesized and labeled with biotin by Takara (the sequence is shown in Figure S6). EMSA was carried out with a gel-shift kit purchased from Active Motif. The competition assay was performed by adding cognate unlabeled oligonucleotides. After incubation, the protein–DNA complexes were separated on a nondenaturing 6% polyacrylamide gel and then transferred to a nitrocellulose membrane (Millipore). The signals were detected using a luminoimage analyzer.

### Streptavidin–agarose pulldown and western blotting

The pulldown assay was performed following a protocol described elsewhere [40], with slight modification. Biotin-labeled allelic probes were incubated with equal amounts of nuclear extract from activated Jurkat cells for 2 hours at room temperature, in the presence of streptavidin–agarose beads (GE Healthcare) and protein inhibitors. The precipitated protein–DNA complex was dissociated from the agarose beads by suspending the pellet in Laemmli sample buffer (Bio-Rad) and heating it. The supernatants were then subjected to SDS–PAGE. The proteins were transferred onto a PVDF membrane (Bio-Rad), blotted with an anti-Ets-1 antibody, and detected with ECL solution (Pierce). To evaluate the Ets-1 protein levels after the transfection of the overexpression vectors or siRNAs, the Jurkat and HeLa cells were lysed in RIPA buffer (Thermo Scientific), and the supernatants were similarly used for immunoblotting. Anti-ETS1, anti-GAPDH, and horseradish-peroxidase-conjugated secondary antibodies were all obtained from Santa Cruz Biotechnology.

### Data analysis

For single SNP analysis, PLINK was used for the basic allelic test and other tests in the patients and the controls [28]. LD patterns were analyzed and displayed with HaploView [41]. Review manager was used to perform meta-analysis. IMPUTE version 2 was used to perform imputation. Other data were analyzed with GraphPad Prism 4 software, version 4.03. The nonparametric Mann–Whitney test was used to compare *miR-146a* expression between the genotype groups, and an unpaired *t* test was used to compare reporter gene activities. Two-tailed *P* values<0.05 were considered to be statistically significant.

## Supporting Information

Figure S1 Illustration of the *miR-146a* genomic region investigated to identify common variants by direct sequencing. (*A*) Genomic structure of the *miR-146a* gene. The two gray boxes represent exons of *pri-miR-146a*, whereas the black box represents pre-*miR-146a*. The gray lines (underneath the genomic structure) indicate the genomic regions that were amplified for sequence analysis. The locations of the five SNPs with minor allele frequencies of >0.01 are shown. TSS, transcription start site. (*B*) Schematic representation of reporter gene constructs driven by various *miR-146a* upstream fragments (*left*) and their corresponding relative luciferase activities in HeLa cells (*right*), under rested (medium) and PMA+Iono-activated conditions. The data shown are means ± SEM and are representative of three independent experiments performed in triplicate.
(TIF)

Figure S2 Linkage disequilibrium of six common SNPs in or upstream of the *miR-146a* promoter. Data are based on 816 SLE patients and 1,080 controls from Shanghai and were analyzed with HaploView.
(PNG)

Figure S3 Plot of −log10 P values for SNPs genotyped in the GWAS spanning 5q33.3 region. Data were from three GWAS on both Asian (*A*) and European population (*B* and *C*). The linkage disequilibrium in the region derived from the Asian GWAS data is shown with r^2^ values as indicated.
(PNG)

Figure S4 Reporter gene activity of constructs containing either rs57095329 allele in different cell lines. Shown are the relative luciferase activities of the two constructs driven by the *miR-146a* promoter containing either rs57095329 allele (A or G) in rested (medium) and activated (with the addition of PMA+Iono for 6 hours) HeLa cells (*A*), steady-state Raji cells (*B*), and steady-state 293T cells (*C*). The data shown are means ± SEM and are representative of three independent experiments performed in triplicate or quadruplicate. * indicates *P*<0.05, ** *P*<0.01, *** *P*<0.0001.
(TIF)

Figure S5 Gel-shift assay of allelic probes with nuclear proteins from different cell lines. Shown is a comparison of the binding affinities of different rs57095329 alleles for the nuclear extracts (N.E.) from rested (medium) or PMA+Iono-activated HeLa cells (*left*), and from PMA+Iono-activated Jurkat cells (*right*). Also shown are the results of a competition assay, which was performed with the addition of 50- to 200-fold unlabeled cognate oligonucleotides. The assays were repeated at least three times.
(TIF)

Figure S6 Predicted binding sites of Ets-1 on the *miR-146a* promoter. (*A*) The 200-nt sequence around rs57095329 (A/G) was used as the input for the Genomatix online tool, which predicted a nearby Ets-1-binding site (indicated by the blue box, with the red letters indicating the core sequence). Also shown is the probe sequence for the EMSA, indicated by the pink line below the sequence. (*B*) Conservation of rs57095329 residue and Ets-1 binding site. Shown is the UCSC Genome Bioinformatics search result by alignment of the sequence around rs5705329 (indicated by the red box) in 7 species.
(TIF)

Figure S7 Analysis of the regulation of *miR-146a* expression by Ets-1 in HeLa cells. (*A*) Comparisons of the *miR-146a* promoter–reporter gene activity after cotransfection of an equal amount of an irrelevant carrier vector or an *ETS1*- or *PBX1*-expressing vector. The data shown are means ± SEM and are representative of three independent experiments performed in triplicate. *** indicates *P*<0.001. (*B*) Comparisons of the *miR-146a* promoter–reporter gene activity after the cotransfection of ETS1 siRNA (siETS1 #1 and siETS1 #2) or a negative control (NC). The data shown are means ± SEM and are representative of three independent experiments performed in triplicate. * indicates *P*<0.05. (*C*) Western blot analysis of Ets-1 levels after the transfection of the indicated expression vectors or siRNA. In the overexpression assay, the cells were collected 24 hours after transfection; in the siRNA-mediated knockdown assay, the cells were collected 48 hours after transfection. GAPDH was used as the loading control.
(TIF)

Figure S8 Effect of ectopic Ets-1 expression on the activity of the allelic *miR-146a* promoter–reporter gene constructs. Reporter gene constructs containing the A or G *miR-146a* sequence were cotransfected into HeLa cells with different amounts of *ETS1*-expressing vector (0, 50, or 200 ng). For these three groups, 200 ng, 150 ng, or 0 ng of an irrelevant carrier vector was cotransfected, respectively, so that equal amounts of total plasmid DNA were used in all groups. The relative luciferase activity was measured 24 hours after transfection (*upper*). Cotransfection of a *PBX1*-expressing vector was used as the negative control. Also shown are the average G/A ratios of the luciferase activity of the allelic constructs (*lower*).
(TIF)

Figure S9 Comparison of *miR-146a* expression levels in healthy individuals with different genotypes of rs2431697 or rs2431099. The horizontal line indicates the mean expression level within each group.
(JPG)

Table S1 A list of the variants identified by the initial sequencing of the *miR-146a* region.
(DOC)

Table S2 Association between the seven common SNPs around *miR-146a* and SLE.
(DOC)

Table S3 Association between the rs57095329 G allele and lupus nephritis.
(DOC)

Table S4 Analysis of OR in case-control groups carrying different numbers of risk alleles of either *miR-146a* or *ETS1* SNP.
(DOC)

Table S5 Conditional analysis of three SNPs in 5q33.3 in SLE cases and controls.
(DOC)

Table S6 Haplotypic association of three SNPs in 5q33.3 with SLE.
(DOC)

Table S7 A list of the primers used for the various assays.
(DOC)

## References

[1] D'Cruz DP, Khamashta MA, Hughes GR (2007). Systemic lupus erythematosus.. Lancet.

[2] Rahman A, Isenberg DA (2008). Systemic lupus erythematosus.. N Engl J Med.

[3] Harley JB, Alarcon-Riquelme ME, Criswell LA, Jacob CO, Kimberly RP (2008). Genome-wide association scan in women with systemic lupus erythematosus identifies susceptibility variants in ITGAM, PXK, KIAA1542 and other loci.. Nat Genet.

[4] Hom G, Graham RR, Modrek B, Taylor KE, Ortmann W (2008). Association of systemic lupus erythematosus with C8orf13-BLK and ITGAM-ITGAX.. N Engl J Med.

[5] Graham RR, Cotsapas C, Davies L, Hackett R, Lessard CJ (2008). Genetic variants near TNFAIP3 on 6q23 are associated with systemic lupus erythematosus.. Nat Genet.

[6] Kozyrev SV, Abelson AK, Wojcik J, Zaghlool A, Linga Reddy MV (2008). Functional variants in the B-cell gene BANK1 are associated with systemic lupus erythematosus.. Nat Genet.

[7] Gateva V, Sandling JK, Hom G, Taylor KE, Chung SA (2009). A large-scale replication study identifies TNIP1, PRDM1, JAZF1, UHRF1BP1 and IL10 as risk loci for systemic lupus erythematosus.. Nat Genet.

[8] Han JW, Zheng HF, Cui Y, Sun LD, Ye DQ (2009). Genome-wide association study in a Chinese Han population identifies nine new susceptibility loci for systemic lupus erythematosus.. Nat Genet.

[9] Yang W, Shen N, Ye DQ, Liu Q, Zhang Y (2010). Genome-wide association study in Asian populations identifies variants in ETS1 and WDFY4 associated with systemic lupus erythematosus.. PLoS Genet.

[10] Crow MK (2008). Collaboration, genetic associations, and lupus erythematosus.. N Engl J Med.

[11] Flesher DL, Sun X, Behrens TW, Graham RR, Criswell LA (2010). Recent advances in the genetics of systemic lupus erythematosus.. Expert Rev Clin Immunol.

[12] Bennett L, Palucka AK, Arce E, Cantrell V, Borvak J (2003). Interferon and granulopoiesis signatures in systemic lupus erythematosus blood.. J Exp Med.

[13] Han GM, Chen SL, Shen N, Ye S, Bao CD (2003). Analysis of gene expression profiles in human systemic lupus erythematosus using oligonucleotide microarray.. Genes Immun.

[14] Baechler EC, Batliwalla FM, Karypis G, Gaffney PM, Ortmann WA (2003). Interferon-inducible gene expression signature in peripheral blood cells of patients with severe lupus.. Proc Natl Acad Sci U S A.

[15] Pascual V, Farkas L, Banchereau J (2006). Systemic lupus erythematosus: all roads lead to type I interferons.. Curr Opin Immunol.

[16] Ronnblom L, Eloranta ML, Alm GV (2006). The type I interferon system in systemic lupus erythematosus.. Arthritis Rheum.

[17] Crow MK (2007). Type I interferon in systemic lupus erythematosus.. Curr Top Microbiol Immunol.

[18] Ronnblom L, Alm GV, Eloranta ML (2009). Type I interferon and lupus.. Curr Opin Rheumatol.

[19] Bartel DP (2004). MicroRNAs: genomics, biogenesis, mechanism, and function.. Cell.

[20] Baltimore D, Boldin MP, O'Connell RM, Rao DS, Taganov KD (2008). MicroRNAs: new regulators of immune cell development and function.. Nat Immunol.

[21] Lodish HF, Zhou B, Liu G, Chen CZ (2008). Micromanagement of the immune system by microRNAs.. Nat Rev Immunol.

[22] Xiao C, Rajewsky K (2009). MicroRNA control in the immune system: basic principles.. Cell.

[23] Tang Y, Luo X, Cui H, Ni X, Yuan M (2009). MicroRNA-146A contributes to abnormal activation of the type I interferon pathway in human lupus by targeting the key signaling proteins.. Arthritis Rheum.

[24] Duan R, Pak C, Jin P (2007). Single nucleotide polymorphism associated with mature miR-125a alters the processing of pri-miRNA.. Hum Mol Genet.

[25] Mencia A, Modamio-Hoybjor S, Redshaw N, Morin M, Mayo-Merino F (2009). Mutations in the seed region of human miR-96 are responsible for nonsyndromic progressive hearing loss.. Nat Genet.

[26] Jazdzewski K, Murray EL, Franssila K, Jarzab B, Schoenberg DR (2008). Common SNP in pre-miR-146a decreases mature miR expression and predisposes to papillary thyroid carcinoma.. Proc Natl Acad Sci U S A.

[27] Taganov KD, Boldin MP, Chang KJ, Baltimore D (2006). NF-kappaB-dependent induction of microRNA miR-146, an inhibitor targeted to signaling proteins of innate immune responses.. Proc Natl Acad Sci U S A.

[28] Purcell S, Neale B, Todd-Brown K, Thomas L, Ferreira MA (2007). PLINK: a tool set for whole-genome association and population-based linkage analyses.. Am J Hum Genet.

[29] Curtale G, Citarella F, Carissimi C, Goldoni M, Carucci N (2010). An emerging player in the adaptive immune response: microRNA-146a is a modulator of IL-2 expression and activation-induced cell death in T lymphocytes.. Blood.

[30] Luo X, Tsai LM, Shen N, Yu D (2010). Evidence for microRNA-mediated regulation in rheumatic diseases.. Ann Rheum Dis.

[31] Xu T, Zhu Y, Wei QK, Yuan Y, Zhou F (2008). A functional polymorphism in the miR-146a gene is associated with the risk for hepatocellular carcinoma.. Carcinogenesis.

[32] Xu B, Feng NH, Li PC, Tao J, Wu D (2010). A functional polymorphism in Pre-miR-146a gene is associated with prostate cancer risk and mature miR-146a expression in vivo.. Prostate.

[33] Delgado-Vega AM, Alarcon-Riquelme ME, Kozyrev SV (2010). Genetic associations in type I interferon related pathways with autoimmunity.. Arthritis Res Ther.

[34] Sigurdsson S, Nordmark G, Goring HH, Lindroos K, Wiman AC (2005). Polymorphisms in the tyrosine kinase 2 and interferon regulatory factor 5 genes are associated with systemic lupus erythematosus.. Am J Hum Genet.

[35] Graham RR, Kozyrev SV, Baechler EC, Reddy MV, Plenge RM (2006). A common haplotype of interferon regulatory factor 5 (IRF5) regulates splicing and expression and is associated with increased risk of systemic lupus erythematosus.. Nat Genet.

[36] Graham RR, Kyogoku C, Sigurdsson S, Vlasova IA, Davies LR (2007). Three functional variants of IFN regulatory factor 5 (IRF5) define risk and protective haplotypes for human lupus.. Proc Natl Acad Sci U S A.

[37] Remmers EF, Plenge RM, Lee AT, Graham RR, Hom G (2007). STAT4 and the risk of rheumatoid arthritis and systemic lupus erythematosus.. N Engl J Med.

[38] Kariuki SN, Kirou KA, MacDermott EJ, Barillas-Arias L, Crow MK (2009). Cutting edge: autoimmune disease risk variant of STAT4 confers increased sensitivity to IFN-alpha in lupus patients in vivo.. J Immunol.

[39] Rullo OJ, Woo JM, Wu H, Hoftman AD, Maranian P (2010). Association of IRF5 polymorphisms with activation of the interferon alpha pathway.. Ann Rheum Dis.

[40] Wu KK (2006). Analysis of protein-DNA binding by streptavidin-agarose pulldown.. Methods Mol Biol.

[41] Barrett JC, Fry B, Maller J, Daly MJ (2005). Haploview: analysis and visualization of LD and haplotype maps.. Bioinformatics.

