# Supplementary figures and images for: A Functional Variant in MicroRNA-146a Promoter Modulates Its Expression and Confers Disease Risk for Systemic Lupus Erythematosus
Source: PLoS Genet. 2011 Jun 30;7(6):e1002128. doi: 10.1371/journal.pgen.1002128 (PMC3128113; doi:10.1371/journal.pgen.1002128)

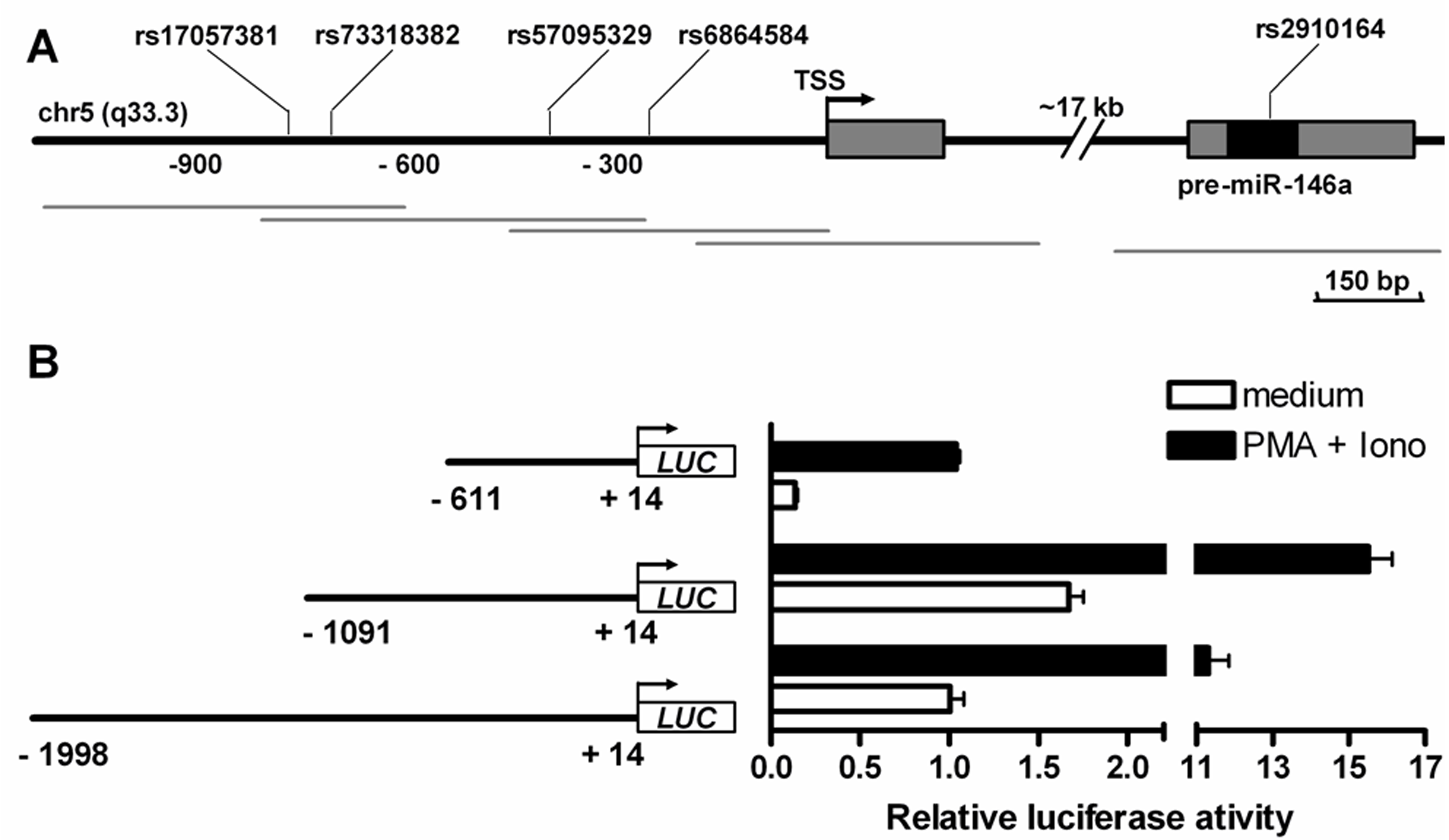

Supplement: Figure S1 — Illustration of the miR-146a genomic region investigated to identify common variants by direct sequencing. (A) Genomic structure of the miR-146a gene. The two gray boxes represent exons of pri-miR-146a, whereas the black box represents pre-miR-146a. The gray lines (underneath the genomic structure) indicate the genomic regions that were amplified for sequence analysis. The locations of the five SNPs with minor allele frequencies of >0.01 are shown. TSS, transcription start site. (B) Schematic representation of reporter gene constructs driven by various miR-146a upstream fragments (left) and their corresponding relative luciferase activities in HeLa cells (right), under rested (medium) and PMA+Iono-activated conditions. The data shown are means ± SEM and are representative of three independent experiments performed in triplicate. (TIF) [file pgen.1002128.s001.tif]

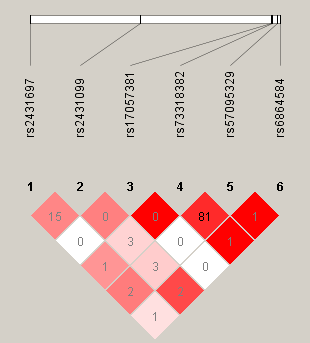

Supplement: Figure S2 — Linkage disequilibrium of six common SNPs in or upstream of the miR-146a promoter. Data are based on 816 SLE patients and 1,080 controls from Shanghai and were analyzed with HaploView. (PNG) [file pgen.1002128.s002.png]

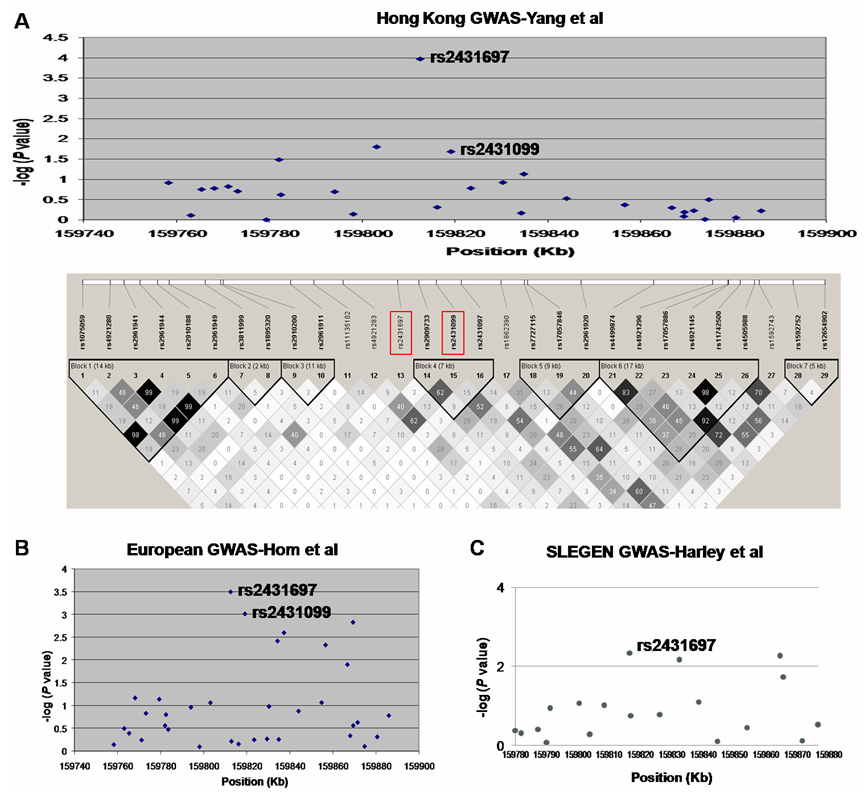

Supplement: Figure S3 — Plot of −log10 P values for SNPs genotyped in the GWAS spanning 5q33.3 region. Data were from three GWAS on both Asian (A) and European population (B and C). The linkage disequilibrium in the region derived from the Asian GWAS data is shown with r2 values as indicated. (PNG) [file pgen.1002128.s003.png]

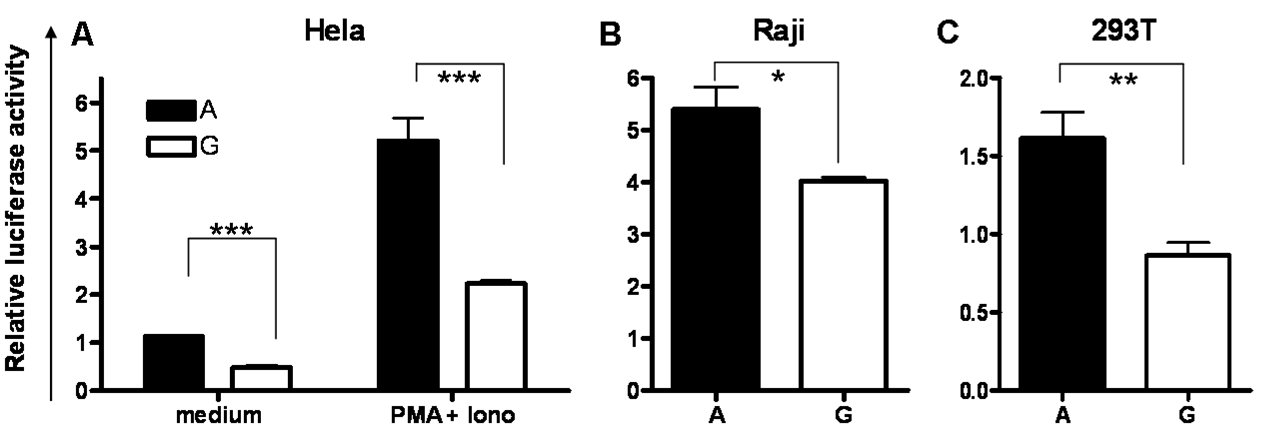

Supplement: Figure S4 — Reporter gene activity of constructs containing either rs57095329 allele in different cell lines. Shown are the relative luciferase activities of the two constructs driven by the miR-146a promoter containing either rs57095329 allele (A or G) in rested (medium) and activated (with the addition of PMA+Iono for 6 hours) HeLa cells (A), steady-state Raji cells (B), and steady-state 293T cells (C). The data shown are means ± SEM and are representative of three independent experiments performed in triplicate or quadruplicate. * indicates P<0.05, ** P<0.01, *** P<0.0001. (TIF) [file pgen.1002128.s004.tif]

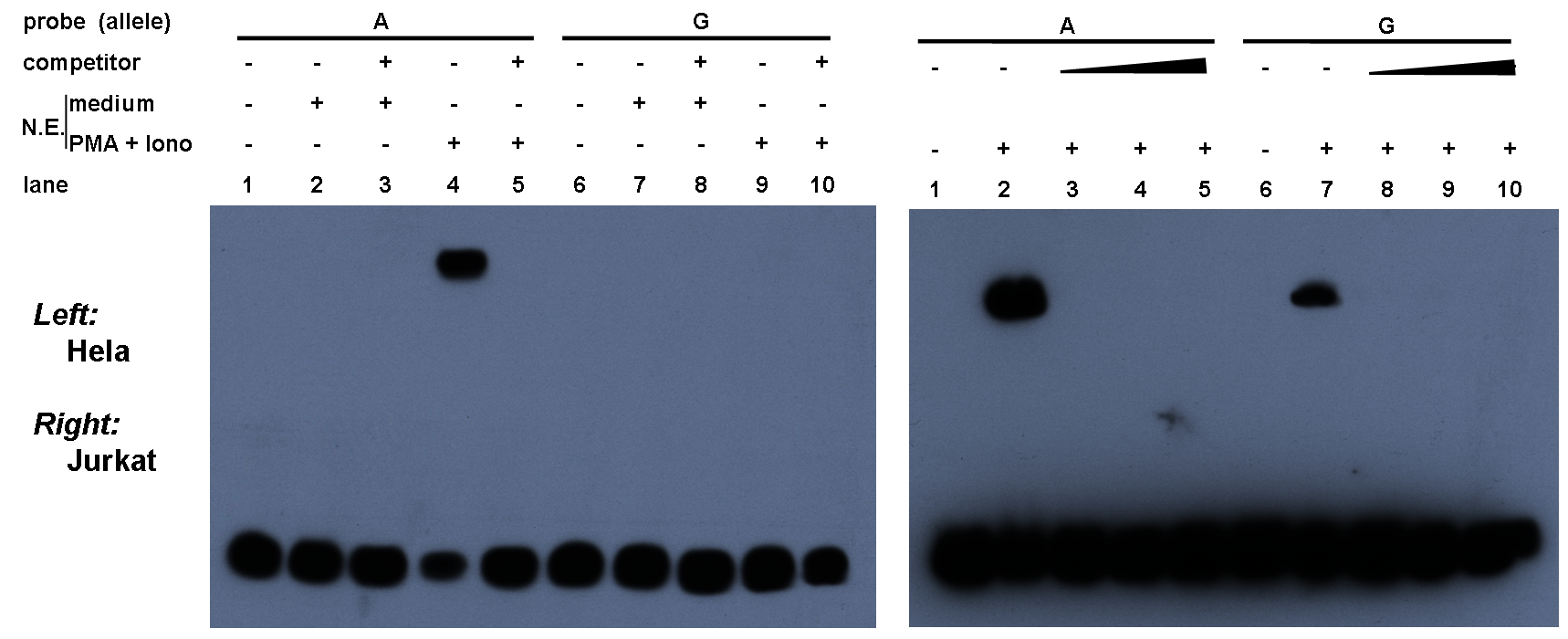

Supplement: Figure S5 — Gel-shift assay of allelic probes with nuclear proteins from different cell lines. Shown is a comparison of the binding affinities of different rs57095329 alleles for the nuclear extracts (N.E.) from rested (medium) or PMA+Iono-activated HeLa cells (left), and from PMA+Iono-activated Jurkat cells (right). Also shown are the results of a competition assay, which was performed with the addition of 50- to 200-fold unlabeled cognate oligonucleotides. The assays were repeated at least three times. (TIF) [file pgen.1002128.s005.tif]

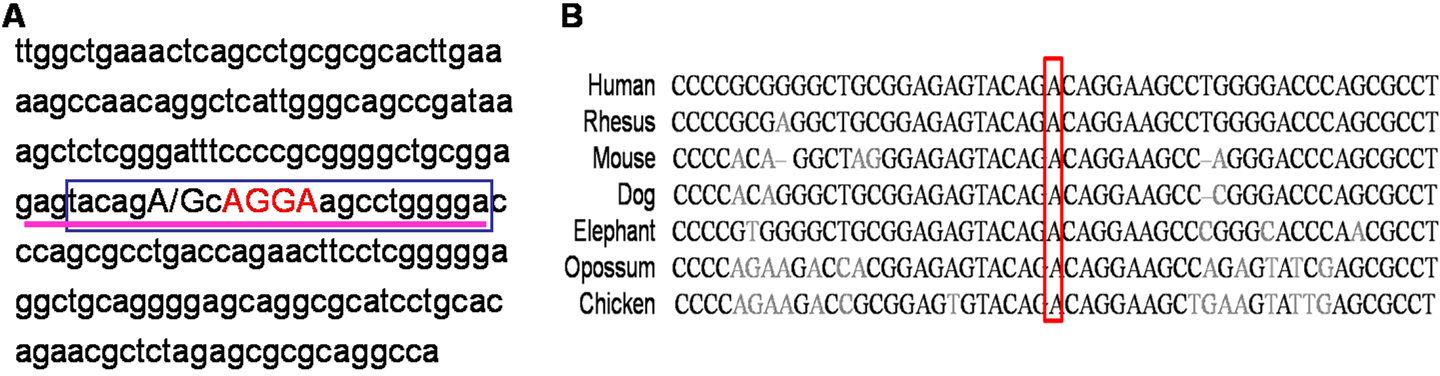

Supplement: Figure S6 — Predicted binding sites of Ets-1 on the miR-146a promoter. (A) The 200-nt sequence around rs57095329 (A/G) was used as the input for the Genomatix online tool, which predicted a nearby Ets-1-binding site (indicated by the blue box, with the red letters indicating the core sequence). Also shown is the probe sequence for the EMSA, indicated by the pink line below the sequence. (B) Conservation of rs57095329 residue and Ets-1 binding site. Shown is the UCSC Genome Bioinformatics search result by alignment of the sequence around rs5705329 (indicated by the red box) in 7 species. (TIF) [file pgen.1002128.s006.tif]

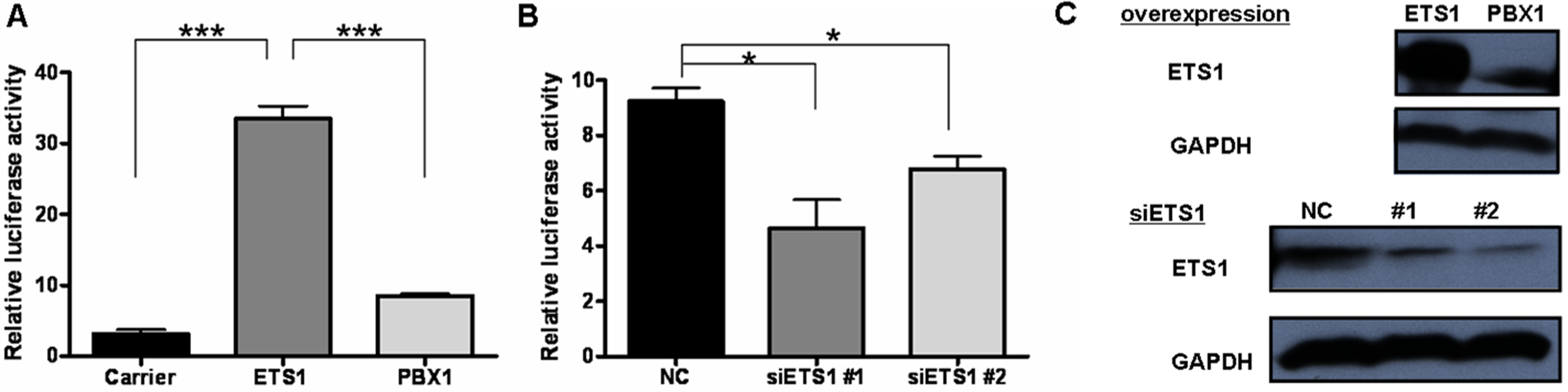

Supplement: Figure S7 — Analysis of the regulation of miR-146a expression by Ets-1 in HeLa cells. (A) Comparisons of the miR-146a promoter–reporter gene activity after cotransfection of an equal amount of an irrelevant carrier vector or an ETS1- or PBX1-expressing vector. The data shown are means ± SEM and are representative of three independent experiments performed in triplicate. *** indicates P<0.001. (B) Comparisons of the miR-146a promoter–reporter gene activity after the cotransfection of ETS1 siRNA (siETS1 #1 and siETS1 #2) or a negative control (NC). The data shown are means ± SEM and are representative of three independent experiments performed in triplicate. * indicates P<0.05. (C) Western blot analysis of Ets-1 levels after the transfection of the indicated expression vectors or siRNA. In the overexpression assay, the cells were collected 24 hours after transfection; in the siRNA-mediated knockdown assay, the cells were collected 48 hours after transfection. GAPDH was used as the loading control. (TIF) [file pgen.1002128.s007.tif]

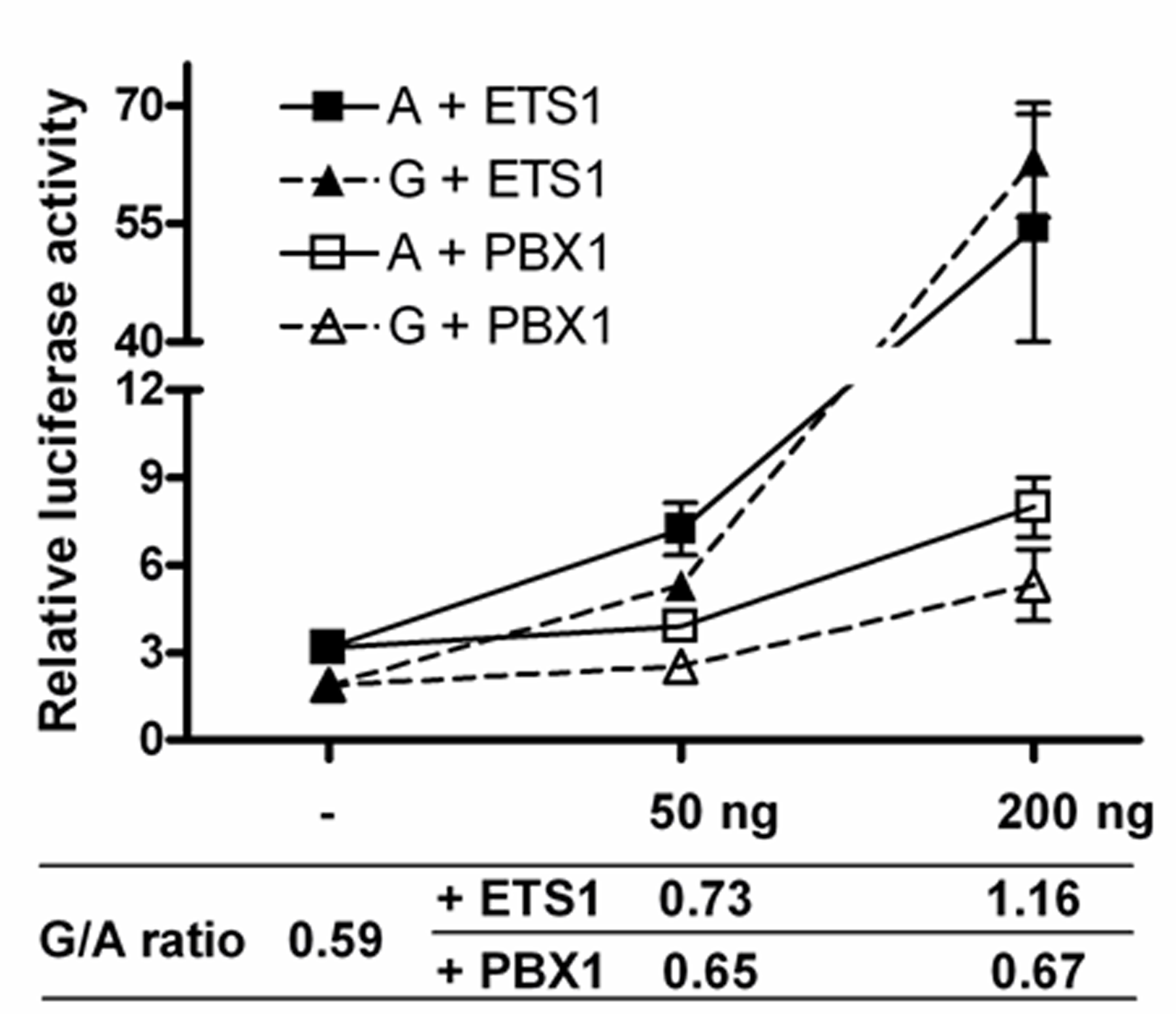

Supplement: Figure S8 — Effect of ectopic Ets-1 expression on the activity of the allelic miR-146a promoter–reporter gene constructs. Reporter gene constructs containing the A or G miR-146a sequence were cotransfected into HeLa cells with different amounts of ETS1-expressing vector (0, 50, or 200 ng). For these three groups, 200 ng, 150 ng, or 0 ng of an irrelevant carrier vector was cotransfected, respectively, so that equal amounts of total plasmid DNA were used in all groups. The relative luciferase activity was measured 24 hours after transfection (upper). Cotransfection of a PBX1-expressing vector was used as the negative control. Also shown are the average G/A ratios of the luciferase activity of the allelic constructs (lower). (TIF) [file pgen.1002128.s008.tif]

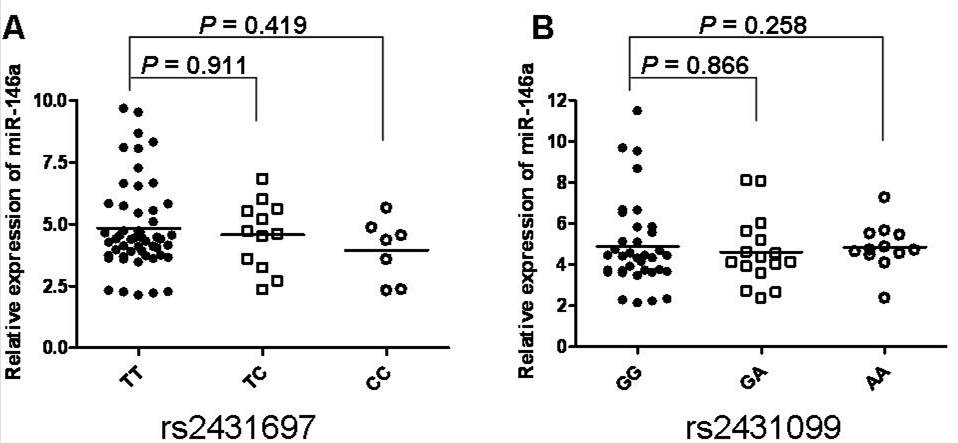

Supplement: Figure S9 — Comparison of miR-146a expression levels in healthy individuals with different genotypes of rs2431697 or rs2431099. The horizontal line indicates the mean expression level within each group. (JPG) [file pgen.1002128.s009.jpg]
