# Supplementary material for: A Functional Variant in MicroRNA-146a Promoter Modulates Its Expression and Confers Disease Risk for Systemic Lupus Erythematosus
Source: PLoS Genet. 2011 Jun 30;7(6):e1002128. doi: 10.1371/journal.pgen.1002128 (PMC3128113; doi:10.1371/journal.pgen.1002128)
Supplement: Table S1 — A list of the variants identified by the initial sequencing of the miR-146a region. (DOC) [file pgen.1002128.s010.doc]

**Table S1. A list of variants identified by initial sequencing of miR-146a region*.**

| **Variant** | **Position** | **Location^†^** | **Allele** | **MAF** | **# Carriers** |
| --- | --- | --- | --- | --- | --- |
| rs35669109 | 159826920 | promoter, nt -891 | -/G | -^‡^ |  |
| miR146asnp1 | 159826989 | promoter, nt -822 | A/G |  | 2 subjects |
| rs17057381 | 159827062 | promoter, nt -749 | T/C | 0.031 |  |
| rs73318382 | 159827121 | promoter, nt -690 | A/C | 0.165 |  |
| rs13157399 | 159827269 | promoter, nt -542 | T/C | -^‡^ |  |
| miR146asnp2 | 159827297 | promoter, nt -514 | A/G |  | 1 subjects |
| rs57095329 | 159827425 | promoter, nt -386 | A/G | 0.183 |  |
| miR146asnp3 | 159827552 | promoter, nt -259 | C/T |  | 1 subjects |
| rs13171086 | 159827554 | promoter, nt -257 | G/A | - ^‡^ |  |
| rs6864584 | 159827563 | promoter, nt -248 | T/C | 0.056 |  |
| rs34359842 | 159827852 | 42^nd^ nt of pri-miR-146a | A/C | - ^‡^ |  |
| rs2910164 | 159844996 | 60^th^ nt of pre-miR-146a | C/G | 0.410 |  |

^*^ The sequencing analysis involves 360 individuals (180 SLE cases and 180 controls) that are all Chinese Han population in Shanghai. ^†^Location is determined with the transcription start site set at 1. ^‡^ SNPs reported in dbSNP Build 130 but monozygous in 360 Chinese Han individuals tested. MAF, minor allele frequency.
