# Supplementary material for: A Functional Variant in MicroRNA-146a Promoter Modulates Its Expression and Confers Disease Risk for Systemic Lupus Erythematosus
Source: PLoS Genet. 2011 Jun 30;7(6):e1002128. doi: 10.1371/journal.pgen.1002128 (PMC3128113; doi:10.1371/journal.pgen.1002128)
Supplement: Table S2 — Association between the seven common SNPs around miR-146a and SLE. (DOC) [file pgen.1002128.s011.doc]

**Table S2. Association of seven common SNPs around miR-146a with SLE*.**

| **SNP** | **Assoc Allele** | **Associated allele frequency** | | **Chi Square** | ***P* value (allele)** | **OR (95% CI)** |
| --- | --- | --- | --- | --- | --- | --- |
|  |  | **Case** | **Control** |  |  |  |
| rs17057381 | C | 0.02 | 0.03 | 1.15 | 0.28 | 0.79(0.52-1.21) |
| rs73318382 | C | 0.20 | 0.16 | 7.90 | 4.93E-3 | 1.27(1.08-1.50) |
| rs57095329 | G | 0.21 | 0.16 | 12.55 | 3.96E-4 | 1.36(1.15-1.62) |
| rs6864584 | C | 0.05 | 0.05 | 0.90 | 0.34 | 0.86(0.64-1.17) |
| rs2910164 | G | 0.41 | 0.40 | 0.61 | 0.44 | 1.05(0.92-1.20) |
| rs2431697 | T | 0.86 | 0.83 | 7.97 | 4.80E-3 | 1.30(1.08-1.56) |
| rs2431099 | G | 0.65 | 0.61 | 5.16 | 2.31E-2 | 1.17(1.02-1.34) |

*****The analysis involves 816 cases and 1080 controls that are all Chinese Han individuals in Shanghai.
