# Supplementary material for: A Functional Variant in MicroRNA-146a Promoter Modulates Its Expression and Confers Disease Risk for Systemic Lupus Erythematosus
Source: PLoS Genet. 2011 Jun 30;7(6):e1002128. doi: 10.1371/journal.pgen.1002128 (PMC3128113; doi:10.1371/journal.pgen.1002128)
Supplement: Table S3 — Association between the rs57095329 G allele and lupus nephritis. (DOC) [file pgen.1002128.s012.doc]

**Table S3. Association of rs57095329 G allele with lupus nephritis.**

| **Sample groups** | **No. of**  **subjects** | **Allele (%)** | | | **Chi Square** | ***P* value (allele)** | | **OR**  **(95% CI)** | |
| --- | --- | --- | --- | --- | --- | --- | --- | --- | --- |
|  |  | **G** | **A** | |  |  |  |  |  |
| **Mainland China** |  |  |  | |  |  | |  | |
| LN^*^ | 806 | 328(21.5) | 1196(78.5) | | 0.67 | 0.415 | | 1.07(0.91-1.25) | |
| Non-LN | 1313 | 507(20.4) | 1973(79.6) | |  |  | |  | |
| **Hong Kong** |  |  |  | |  |  | |  | |
| LN | 436 | 211(24.5) | 651(75.5) | | 2.15 | 0.143 | | 1.11(0.91-1.36) | |
| Non-LN | 705 | 312(22.6) | 1064(77.4) | |  |  | |  | |
| **Bangkok** |  |  |  | |  |  | |  | |
| LN | 362 | 210(30.3) | 484(69.7) | | 2.02 | 0.156 | | 1.30(0.90-1.87) | |
| Non-LN | 102 | 48(25.0) | 144(75.0) | |  |  | |  | |
| **Combined^†^** |  | **P(R)** | | **OR(R)** | | | **Q** | | **I** |
| LN | 1604 | 0.093 | | 1.105 | | | 0.614 | | 0.00 |
| Non-LN | 2120 |  | |  | | |  | |  |

**^*^**LN, lupus nephritis. **^†^**A joint analysis is performed for combined samples. P(R) is the *P* value assuming a random-effects model in meta-analysis; OR(R) is the odds ratio assuming a random-effects model; Q is the P value for between study heterogeneity by Cochran’s Q statistic and I is I^2 heterogeneity index (0-100).
