# Supplementary material for: A Functional Variant in MicroRNA-146a Promoter Modulates Its Expression and Confers Disease Risk for Systemic Lupus Erythematosus
Source: PLoS Genet. 2011 Jun 30;7(6):e1002128. doi: 10.1371/journal.pgen.1002128 (PMC3128113; doi:10.1371/journal.pgen.1002128)
Supplement: Table S4 — Analysis of OR in case-control groups carrying different numbers of risk alleles of either miR-146a or ETS1 SNP. (DOC) [file pgen.1002128.s013.doc]

**Table S4. Analysis of OR in case-control groups carrying different numbers of risk alleles of either *miR-146a* or *ETS1* SNP.**

| **Number of risk allele** | **rs57095329**  **-rs1128334** | **Case** | **Ctrl** | ***P*** | **OR (95% CI)** |
| --- | --- | --- | --- | --- | --- |
| 4 | GG-AA | 19 | 6 | 2.7E-04 | 4.79 (1.9-12.09) |
|  |  |  |  |  |  |
| 3 | GG-GA | 59 | 44 | 5.7E-04 | 2.03 (1.35-3.05) |
| 3 | GA-AA | 122 | 87 | 6.6E-07 | 2.12 (1.57-2.86) |
|  |  |  |  |  |  |
| 2 | GG-GG | 45 | 39 | 0.013 | 1.75 (1.12-2.73) |
| 2 | AA-AA | 164 | 157 | 3.2E-04 | 1.58 (1.23-2.03) |
|  |  |  |  |  |  |
| 2 | GA-GA | 286 | 348 | 0.031 | 1.24 (1.02-1.52) |
|  |  |  |  |  |  |
| 1 | GA-GG | 246 | 297 | 0.033 | 1.25 (1.02-1.54) |
| 1 | AA-GA | 560 | 730 | 0.075 | 1.16 (0.99-1.37) |
|  |  |  |  |  |  |
| 0 | AA-GG | 435 | 658 | Reference | 1.00 |
