# Supplementary material for: A Functional Variant in MicroRNA-146a Promoter Modulates Its Expression and Confers Disease Risk for Systemic Lupus Erythematosus
Source: PLoS Genet. 2011 Jun 30;7(6):e1002128. doi: 10.1371/journal.pgen.1002128 (PMC3128113; doi:10.1371/journal.pgen.1002128)
Supplement: Table S5 — Conditional analysis of three SNPs in 5q33.3 in SLE cases and controls. (DOC) [file pgen.1002128.s014.doc]

**Table S5. Conditional analysis of three SNPs in 5q33.3 with SLE in Chinese samples.**

| SNP | Allele | MAF (%) | | *P* | *P* after conditioning on | | |
| --- | --- | --- | --- | --- | --- | --- | --- |
|  |  | Case | Ctrl |  | rs2431697 | rs2431099 | rs57095329 |
| rs2431697 | C | 13.6 | 17.4 | 0.0024 | - | 0.032 | 0.0096 |
| rs2431099 | A | 34.8 | 39.0 | 0.012 | 0.49 | - | 0.040 |
| rs57095329 | G | 21.0 | 16.1 | 0.00023 | 0.00099 | 0.0042 | - |
