# Supplementary material for: A Functional Variant in MicroRNA-146a Promoter Modulates Its Expression and Confers Disease Risk for Systemic Lupus Erythematosus
Source: PLoS Genet. 2011 Jun 30;7(6):e1002128. doi: 10.1371/journal.pgen.1002128 (PMC3128113; doi:10.1371/journal.pgen.1002128)
Supplement: Table S6 — Haplotypic association of three SNPs in 5q33.3 with SLE. (DOC) [file pgen.1002128.s015.doc]

**Table S6. Haplotypic association of three SNPs in 5q33.3 with SLE.**

|  | rs2431697 | rs2431099 | rs57095329 | Freq (%) | | OR | *Spec P* | *P** |
| --- | --- | --- | --- | --- | --- | --- | --- | --- |
|  |  |  |  | Case | Ctrl |  |  |  |
| H1 | T | G | ***G*** | 17.7 | 13.2 | 1.49 | **6.60E-05** | **0.37** |
| H2 | ***C*** | ***A*** | A | 10.4 | 13.7 | 0.70 | **0.0017** | 0.035 |
| H3 | T | G | A | 45.3 | 45.5 | 1.00 | 0.95 | 4.54E-04 |
| H4 | T | ***A*** | A | 21.3 | 22.4 | 0.93 | 0.43 | 6.01E-04 |
| H5 | T | ***A*** | ***G*** | 2.6 | 2.2 | 1.48 | 0.23 | 8.83E-04 |
| H6 | ***C*** | G | A | 2.7 | 3.0 | 0.83 | 0.42 | 6.08E-04 |

*P**: Omnibus haplotypic association (0.0011) after conditioning on two SLE-associated haplotypes, respectively.
