# Supplementary material for: A Functional Variant in MicroRNA-146a Promoter Modulates Its Expression and Confers Disease Risk for Systemic Lupus Erythematosus
Source: PLoS Genet. 2011 Jun 30;7(6):e1002128. doi: 10.1371/journal.pgen.1002128 (PMC3128113; doi:10.1371/journal.pgen.1002128)
Supplement: Table S7 — A list of the primers used for the various assays. (DOC) [file pgen.1002128.s016.doc]

**Table S4. A list of primers used for various assays.**

| **Assay** | **Forward (5’ – 3’)** | **Reverse (5’ – 3’)** | **size(bp)** |
| --- | --- | --- | --- |
| **cloning** |  |  |  |
| -1,998 to +14 | gtcacgcgtattctggctccaagcat | gtgagatctggagagactcttat | 2,012 |
| -1,091 to +14 | gtgacgcgtgaccaaggaaagg | gtgagatctggagagactcttat | 1,105 |
| -611 to +14 | gtgacgcgtgtatctgcagcaat | gtgagatctggagagactcttat | 625 |
| **sequencing** |  |  |  |
| promoter | gaccaaggaaaggaagctat | cttgtttcattgctgcagat | 502 |
| promoter | taggtgcccattaaaatttag | tcttgcagcacgtgtcag | 535 |
| promoter | attgggcagccgataaa | cttatttgctggggtagagg | 443 |
| exon1 | gcggagagaagctga cact | gcctgctgcctctcaaac | 475 |
| precursor | ggtctcctccagatgtttata | atcattcatttagctacttgg | 452 |
| **real-time PCR** |  |  |  |
| pri-miR-146a | gccgattggagtggtaaac | aagacccctcgttgcagt | 177 |
| RPL13A | cctggaggagaagaggaaagaga | ttgaggacctctgtgtatttgtcaa | 307 |
